# Tris(3-nitro­pentane-2,4-dionato-κ^2^ *O*,*O*′)­cobalt(III)

**DOI:** 10.1107/S160053681200668X

**Published:** 2012-02-24

**Authors:** Dean H. Johnston, Jack T. Brangham, Christopher D. Rapp

**Affiliations:** a Department of Chemistry, Otterbein University, Westerville, OH 43081, USA

## Abstract

The structure of the title compound, [Co(C_5_H_6_NO_4_)_3_], consists of a Co^III^ ion octahedrally coordinated by three bidentate 3-nitro­pentane-2,4-dionate ligands. The complex was prepared *via* the nitration of tris­(2,4-penta­nedionato-κ^2^ *O*,*O*′)cobalt(III) with a solution of copper(II) nitrate in glacial acetic acid. The central C atom and the nitro group of one 3-nitro­pentane-2,4-dionate ligand are disordered over two positions with an occupancy ratio of 0.848 (4):0.152 (4). A second nitro group is also disordered over two orientations with an occupancy ratio of 0.892 (7):0.108 (7). Two of the ligand methyl groups form C—H⋯O inter­actions with two different nitro groups to form chains running along the *c* axis. Additional C—H⋯O inter­actions are found between ligand methyl groups and the cobalt-bound O atoms, also resulting in the formation of chains along the *c* axis.

## Related literature

For the preparation of derivatized tris­(2,4-penta­nedionato) metal complexes, see: Collman *et al.* (1962 ▶, 1963 ▶); Collman (1965 ▶); Schirado *et al.* (1971 ▶); James (1974 ▶); Shalhoub (1980 ▶). For spectroscopic properties of the title compound, see: Singh & Sahai (1967 ▶, 1968 ▶); Larsson & Eskilsson (1969 ▶); Fleming & Thorton (1973 ▶, 1975 ▶); Tsiamis *et al.* (1987 ▶). For crystallographic studies of related compounds, see: Appleton *et al.* (1992 ▶); Abrahams *et al.* (1998 ▶); Tsiamis *et al.* (1998 ▶); von Chrzanowski *et al.* (2007 ▶). For a review of graph-set analysis of hydrogen-bonding patterns, see: Bernstein *et al.* (1995 ▶).
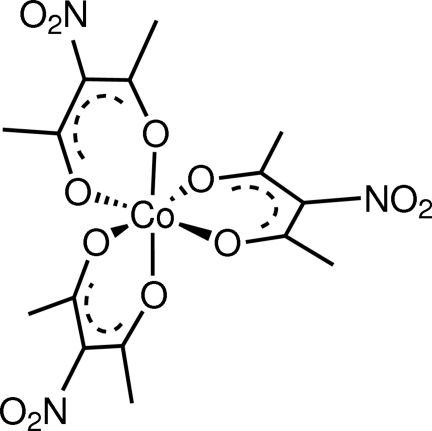


## Experimental

### 

#### Crystal data

[Co(C_5_H_6_NO_4_)_3_]
*M* *_r_* = 491.25
Tetragonal, 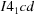

*a* = 32.7078 (18) Å
*c* = 7.4976 (6) Å
*V* = 8020.9 (9) Å^3^
*Z* = 16
Mo *K*α radiation
μ = 0.93 mm^−1^
*T* = 200 K
0.48 × 0.40 × 0.32 mm

#### Data collection

Bruker SMART X2S benchtop diffractometer
Absorption correction: multi-scan (*SADABS*; Bruker, 2009 ▶) *T*_min_ = 0.665, *T*_max_ = 0.756
24724 measured reflections
3393 independent reflections
3151 reflections with *I* > 2σ(*I*)
*R*_int_ = 0.036

#### Refinement

*R*[*F* ^2^ > 2σ(*F* ^2^)] = 0.026
*wR*(*F* ^2^) = 0.061
*S* = 1.04
3393 reflections
330 parameters
159 restraints
H-atom parameters constrained
Δρ_max_ = 0.20 e Å^−3^
Δρ_min_ = −0.19 e Å^−3^
Absolute structure: Flack (1983 ▶), 1466 Friedel pairs
Flack parameter: 0.003 (12)

### 

Data collection: *GIS* (Bruker, 2009 ▶); cell refinement: *SAINT* (Bruker, 2009 ▶); data reduction: *SAINT*; program(s) used to solve structure: *SHELXS97* (Sheldrick, 2008 ▶); program(s) used to refine structure: *SHELXL97* (Sheldrick, 2008 ▶) and *OLEX2* (Dolomanov *et al.*, 2009 ▶); molecular graphics: *PLATON* (Spek, 2009 ▶), *Mercury* (Macrae *et al.*, 2008 ▶) and *POV-RAY* (Cason, 2004 ▶); software used to prepare material for publication: *publCIF* (Westrip, 2010 ▶).

## Supplementary Material

Crystal structure: contains datablock(s) I, global. DOI: 10.1107/S160053681200668X/zl2451sup1.cif

Structure factors: contains datablock(s) I. DOI: 10.1107/S160053681200668X/zl2451Isup2.hkl

Supplementary material file. DOI: 10.1107/S160053681200668X/zl2451Isup3.mol

Additional supplementary materials:  crystallographic information; 3D view; checkCIF report

## Figures and Tables

**Table 1:** Hydrogen-bond geometry (Å, °)

*D*—H⋯*A*	*D*—H	H⋯*A*	*D*⋯*A*	*D*—H⋯*A*
C1—H1*B*⋯O7^i^	0.98	2.33	3.087 (4)	134
C11—H11*B*⋯O12^ii^	0.98	2.55	3.240 (4)	128
C10—H10*C*⋯O5^iii^	0.98	2.46	3.433 (3)	176
C15—H15*C*⋯O3^iv^	0.98	2.57	3.542 (4)	174

Symmetry codes: (i) 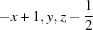; (ii) 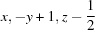; (iii) 
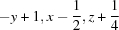
; (iv) 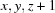.

**Fig. 1.**
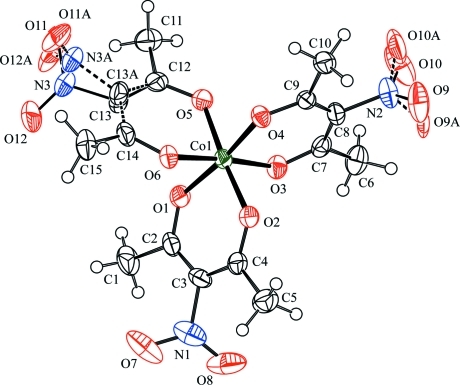
The molecular structure of the title compound showing the atom labeling scheme and drawn with 50% probability displacement ellipsoids for non-H atoms.

**Fig. 2.**
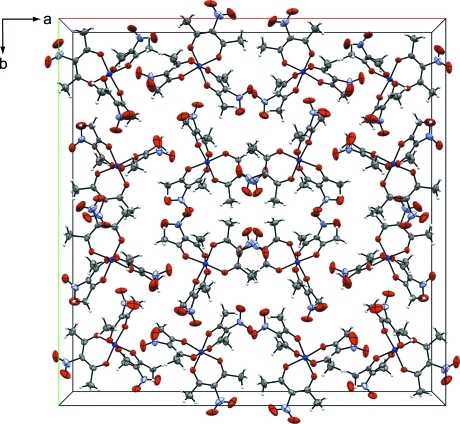
The packing of the title compound viewed along the c axis. The minor components of the disordered ligands are not shown.

**Fig. 3.**
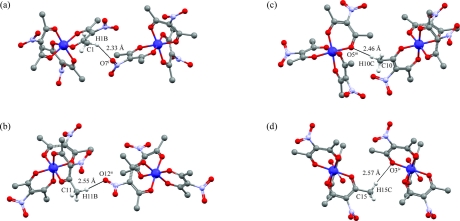
Hydrogen-bond interactions in the title compound. The C—H···O contacts are shown with dashed lines. (a) The C1—H1B···O7i contact, (b) the C11—H11B···O12ii contact, (c) the C10—H10C···O5iii contact, (d) the C15—H15C···O3iv contact. [Symmetry codes: (i) -x + 1, y, z - 1/2; (ii) x, -y + 1, z - 1/2; (iii) -y + 1, x - 1/2, z + 1/4; (iv) x, y, z + 1.]

**Fig. 4.**
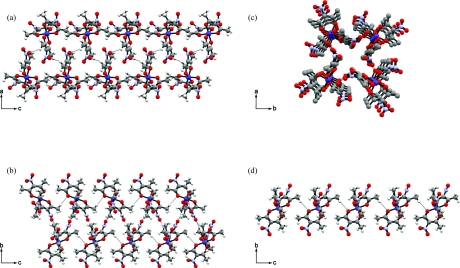
The four different types of C(6) hydrogen-bonded chains formed by the title compound. The C—H···O contacts are shown with dashed lines. (a) The C1—H1B···O7i chain, viewed along the b axis, (b) the C11—H11B···O12ii chain, viewed along the a axis, (c) the C10—H10C···O5iii chain, viewed along the c axis (d) the C15—H15C···O3iv chain, viewed along the a axis. [Symmetry codes: (i) -x + 1, y, z - 1/2; (ii) x, -y + 1, z - 1/2; (iii) -y + 1, x - 1/2, z + 1/4; (iv) x, y, z + 1.]

## supplementary crystallographic information

### Comment

The electrophilic substitution chemistry of the 2,4-pentanedionato (acetylacetonate, or acac) ligand has been studied for many years (Collman, *et al.*, 1962; Collman, *et al.*, 1963; Collman, 1965; Schirado, *et al.*, 1971), but relatively few of these derivatives have been studied crystallographically, especially for the tri-substituted complexes. The nitro derivative of the cobalt complex is readily prepared and its synthesis and characterization have been described as part of several educational laboratory activities (James, 1974; Shalhoub, 1980).

The average cobalt-oxygen bond length in the title compound is 1.869 (4) Å, slightly shorter than the average cobalt-oxygen bond length observed for the [Co(acac)_3_] complex determined at a similar temperature (von Chrzanowski, *et al.*, 2007). All three nitro groups are twisted with respect to their 2,4-pentanedionato ligands (Fig. 1), forming angles of 49.3 (1), 59.3 (2), and 50.3 (2) degrees for the major components and 67.2 (2) and 51.6 (8) degrees for the minor disorder components. These are similar to the angle of 50.7 degrees observed for the mono-nitro cobalt complex (Appleton *et al.*, 1992). The disorder in the positioning of one chelate ring has been observed previously (as large thermal parameters) for analogous complexes of cobalt and manganese (Appleton *et al.*, 1992).

Analysis of packing (Fig. 2) and close contacts shows two different types of C—H···O interactions (Table 1). The first type, shown in Figure 3(*a*) and 3(*b*), forms between methyl group hydrogen atoms and the nitro group on an adjacent molecule. The second type of C—H···O, shown in Figure 3(*c*) and 3(*d*), forms between methyl group hydrogen atoms and the cobalt-bound oxygen atom on an adjacent molecule. This second type of interaction is commonly seen in 2,4-pentanedionato complexes (von Chrzanowski *et al.*, 2007). These hydrogen-bonding interactions result in the formation of four different types of *C*(6) chains (Bernstein *et al.*, 1995), shown in Figure 4(*a*) through 4(*d*). In all four cases, the primary direction of the chain is along the *c* axis.

### Experimental

The complex was prepared according to the procedure of Collman *et al.* (1963). Approximately 5.37 g (0.023 mol) of finely ground copper(II) nitrate trihydrate was mixed with 100 ml (1.06 mol) of acetic anhydride. Cobalt(III) acetylacetonate (2.5 g, 0.0070 mol) was added to the mixture and stirred with cooling for approximately two hours. A combination of water (300 ml), ice (300 g), and sodium acetate (7.5 g, 0.055 mol) was then added and the mixture was stirred for an additional two hours. The dark-green precipitate was vacuum filtered and washed with water and cold ethanol. The crude product was recrystallized from boiling chloroform and hot ethanol. The final product consisted of large, dark green crystals that were obtained in an overall yield of 62% (2.14 g).

The IR spectrum (ATR cell) displayed strong peaks at 1561 cm^-1^ (ν_ring_), 1518 cm^-1^ (ν_as_, NO_2_), 1341 cm^-1^ (ν_s_, NO_2_), and 825 cm^-1^ (δ_C—H_). Raman spectra (532 nm excitation) gave strong peaks at 1345 cm^-1^ (ν_s_, NO_2_), 828 cm^-1^ (δ_C—H_), 470 cm^-1^ and 450 cm^-1^ (ν_Co—O_).

### Refinement

All hydrogen atoms were located in the difference map and refined with the atom positions constrained to an ideal tetrahedron with C—H distances of 0.98 Å. A riding model was used for all hydrogen atoms with *U*_iso_(H) = 1.5 times *U*_iso_(C).

One of the 3-nitropentane-2,4-dionato ligands was modeled as disordered over two positions for four atoms, C13/C13A, N3/N3A, O11/O11A, and O12/O12A and refined to give an occupancy ratio of 0.848 (4):0.152 (4). Carbon-carbon distances between similar atoms in the disordered ligand were restrained to be similar within a standard deviation of 0.02 Å. The nitro groups and their respective carbon atoms (C13/N3/O11/O12, C13A/N3A/O11A/O12A) were restrained to lie in a common plane, as were atoms C12, C13A, C14 and N3A. The anisotropic displacement parameters for the atom pairs N3/N3A and C13/C13A were constrained to be the same. The nitro group on a second ligand (N2, O9, O10) was modeled as a disordered group over two orientations and refined to give an occupancy ratio of 0.892 (7):0.108 (7).

Anisotropic displacement parameters were restrained to be similar (standard deviations of 0.01 Å^2^, 0.02 Å^2^) for 1,2 and 1,3-bonded atoms and approximately isotropic (standard deviation of 0.1 Å^2^) for all disordered oxygen atoms. Anisotropic displacement parameters were also restrained to be similar (with a standard deviation of 0.01 Å^2^) for all atoms within the disordered nitro groups.

### Crystal data

**Table:**

[Co(C_5_H_6_NO_4_)_3_]	*D*_x_ = 1.627 Mg m^−^^3^
*M**_r_* = 491.25	Mo *K*α radiation, λ = 0.71073 Å
Tetragonal, *I*4_1_*c**d*	Cell parameters from 8816 reflections
*a* = 32.7078 (18) Å	θ = 2.5–24.6°
*c* = 7.4976 (6) Å	µ = 0.93 mm^−^^1^
*V* = 8020.9 (9) Å^3^	*T* = 200 K
*Z* = 16	Block, green
*F*(000) = 4032	0.48 × 0.40 × 0.32 mm

### Data collection

**Table:**

Bruker SMART X2S benchtop diffractometer	3393 independent reflections
Radiation source: fine-focus sealed tube	3151 reflections with *I* > 2σ(*I*)
Doubly curved silicon crystal monochromator	*R*_int_ = 0.036
Detector resolution: 8.3330 pixels mm^-1^	θ_max_ = 25.1°, θ_min_ = 2.5°
φ and ω scans	*h* = −38→38
Absorption correction: multi-scan (*SADABS*; Bruker, 2009)	*k* = −38→34
*T*_min_ = 0.665, *T*_max_ = 0.756	*l* = −7→8
24724 measured reflections	

### Refinement

**Table:**

Refinement on *F*^2^	Secondary atom site location: difference Fourier map
Least-squares matrix: full	Hydrogen site location: difference Fourier map
*R*[*F*^2^ > 2σ(*F*^2^)] = 0.026	H-atom parameters constrained
*wR*(*F*^2^) = 0.061	*w* = 1/[σ^2^(*F*_o_^2^) + (0.0316*P*)^2^ + 2.8577*P*] where *P* = (*F*_o_^2^ + 2*F*_c_^2^)/3
*S* = 1.04	(Δ/σ)_max_ = 0.001
3393 reflections	Δρ_max_ = 0.20 e Å^−^^3^
330 parameters	Δρ_min_ = −0.19 e Å^−^^3^
159 restraints	Absolute structure: Flack (1983), 1466 Friedel pairs
Primary atom site location: structure-invariant direct methods	Flack parameter: 0.003 (12)

### Special details

**Table:**

Geometry. All e.s.d.'s (except the e.s.d. in the dihedral angle between two l.s. planes) are estimated using the full covariance matrix. The cell e.s.d.'s are taken into account individually in the estimation of e.s.d.'s in distances, angles and torsion angles; correlations between e.s.d.'s in cell parameters are only used when they are defined by crystal symmetry. An approximate (isotropic) treatment of cell e.s.d.'s is used for estimating e.s.d.'s involving l.s. planes.
Refinement. Refinement of *F*^2^ against ALL reflections. The weighted *R*-factor *wR* and goodness of fit *S* are based on *F*^2^, conventional *R*-factors *R* are based on *F*, with *F* set to zero for negative *F*^2^. The threshold expression of *F*^2^ > σ(*F*^2^) is used only for calculating *R*-factors(gt) *etc*. and is not relevant to the choice of reflections for refinement. *R*-factors based on *F*^2^ are statistically about twice as large as those based on *F*, and *R*- factors based on ALL data will be even larger.

### Fractional atomic coordinates and isotropic or equivalent isotropic displacement parameters (Å^2^)

**Table:**

	*x*	*y*	*z*	*U*_iso_*/*U*_eq_	Occ. (<1)
Co1	0.623604 (9)	0.363125 (9)	0.26098 (5)	0.02596 (9)	
O1	0.60216 (5)	0.41367 (5)	0.1946 (3)	0.0344 (4)	
O2	0.57311 (5)	0.33960 (5)	0.3185 (2)	0.0328 (4)	
C1	0.55984 (10)	0.46990 (8)	0.1698 (5)	0.0506 (8)	
H1A	0.5562	0.4848	0.2820	0.076*	
H1B	0.5357	0.4737	0.0944	0.076*	
H1C	0.5841	0.4803	0.1076	0.076*	
C2	0.56542 (8)	0.42488 (7)	0.2088 (4)	0.0335 (6)	
C3	0.53378 (7)	0.39769 (8)	0.2526 (4)	0.0343 (6)	
C4	0.53853 (8)	0.35653 (8)	0.3056 (3)	0.0321 (6)	
C5	0.50367 (9)	0.32949 (9)	0.3579 (4)	0.0457 (7)	
H5A	0.4921	0.3167	0.2511	0.069*	
H5B	0.4826	0.3458	0.4174	0.069*	
H5C	0.5135	0.3082	0.4394	0.069*	
N1	0.49174 (8)	0.41312 (8)	0.2502 (4)	0.0537 (7)	
O7	0.48554 (8)	0.44588 (8)	0.3244 (4)	0.0868 (9)	
O8	0.46521 (7)	0.39291 (9)	0.1762 (4)	0.0736 (8)	
O3	0.61607 (5)	0.34505 (5)	0.0263 (3)	0.0323 (4)	
O4	0.64932 (5)	0.31545 (5)	0.3405 (2)	0.0292 (4)	
C6	0.61730 (9)	0.30447 (8)	−0.2281 (4)	0.0458 (7)	
H6A	0.5967	0.3245	−0.2651	0.069*	
H6B	0.6063	0.2768	−0.2436	0.069*	
H6C	0.6419	0.3077	−0.3014	0.069*	
C7	0.62781 (7)	0.31108 (7)	−0.0377 (3)	0.0308 (6)	
C9	0.65864 (6)	0.28479 (7)	0.2463 (4)	0.0271 (5)	
C10	0.68264 (8)	0.25280 (8)	0.3421 (4)	0.0360 (6)	
H10A	0.6953	0.2648	0.4484	0.054*	
H10B	0.7040	0.2421	0.2631	0.054*	
H10C	0.6644	0.2305	0.3778	0.054*	
C8	0.64852 (7)	0.28190 (7)	0.0659 (3)	0.0303 (6)	
N2	0.66001 (8)	0.24416 (7)	−0.0254 (4)	0.0478 (6)	
O9	0.64699 (12)	0.21173 (8)	0.0305 (5)	0.0709 (11)	0.892 (7)
O10	0.68344 (13)	0.24671 (10)	−0.1541 (5)	0.0844 (13)	0.892 (7)
O9A	0.6323 (5)	0.2214 (6)	−0.064 (4)	0.047 (5)	0.108 (7)
O10A	0.6956 (5)	0.2340 (7)	−0.042 (5)	0.071 (6)	0.108 (7)
O5	0.67470 (5)	0.38351 (5)	0.1938 (2)	0.0318 (4)	
O6	0.62659 (5)	0.38295 (5)	0.4944 (3)	0.0309 (4)	
C11	0.72986 (8)	0.42775 (9)	0.1701 (4)	0.0436 (7)	
H11A	0.7548	0.4176	0.2274	0.065*	
H11B	0.7301	0.4577	0.1704	0.065*	
H11C	0.7287	0.4178	0.0468	0.065*	
C12	0.69317 (7)	0.41265 (7)	0.2705 (4)	0.0330 (6)	
C14	0.64901 (8)	0.41223 (7)	0.5448 (4)	0.0358 (7)	
C15	0.64112 (10)	0.42602 (9)	0.7323 (4)	0.0495 (8)	
H15A	0.6184	0.4456	0.7331	0.074*	
H15B	0.6657	0.4391	0.7801	0.074*	
H15C	0.6340	0.4023	0.8062	0.074*	
C13	0.67903 (11)	0.42915 (11)	0.4312 (5)	0.0372 (8)	0.848 (4)
N3	0.69807 (11)	0.46657 (10)	0.4996 (6)	0.0564 (9)	0.848 (4)
O11	0.73566 (11)	0.46798 (13)	0.5000 (8)	0.0929 (16)	0.848 (4)
O12	0.67605 (8)	0.49417 (7)	0.5453 (5)	0.0709 (11)	0.848 (4)
C13A	0.6847 (5)	0.4257 (5)	0.4490 (19)	0.0372 (8)	0.152 (4)
N3A	0.7157 (6)	0.4544 (5)	0.521 (3)	0.0564 (9)	0.152 (4)
O11A	0.7310 (5)	0.4489 (5)	0.665 (2)	0.073 (5)	0.152 (4)
O12A	0.7248 (7)	0.4836 (5)	0.433 (3)	0.073 (5)	0.152 (4)

### Atomic displacement parameters (Å^2^)

**Table:**

	*U*^11^	*U*^22^	*U*^33^	*U*^12^	*U*^13^	*U*^23^
Co1	0.02761 (17)	0.02064 (16)	0.02963 (16)	0.00184 (12)	−0.00204 (16)	−0.00131 (15)
O1	0.0348 (10)	0.0206 (8)	0.0479 (12)	0.0007 (7)	−0.0062 (8)	0.0027 (8)
O2	0.0318 (9)	0.0250 (9)	0.0416 (11)	−0.0002 (7)	−0.0010 (8)	−0.0023 (7)
C1	0.068 (2)	0.0282 (15)	0.056 (2)	0.0156 (14)	−0.0166 (17)	−0.0017 (13)
C2	0.0436 (15)	0.0251 (13)	0.0318 (15)	0.0094 (11)	−0.0087 (12)	−0.0073 (11)
C3	0.0296 (13)	0.0407 (14)	0.0325 (15)	0.0138 (11)	−0.0020 (13)	−0.0048 (13)
C4	0.0341 (14)	0.0344 (14)	0.0280 (16)	−0.0011 (11)	−0.0006 (11)	−0.0076 (11)
C5	0.0364 (15)	0.0536 (18)	0.0470 (18)	−0.0064 (14)	0.0034 (14)	−0.0052 (15)
N1	0.0492 (17)	0.0667 (16)	0.0451 (15)	0.0238 (13)	0.0066 (15)	0.0083 (15)
O7	0.087 (2)	0.0720 (16)	0.102 (2)	0.0464 (15)	0.0249 (16)	−0.0003 (15)
O8	0.0335 (12)	0.116 (2)	0.0715 (19)	0.0089 (14)	−0.0101 (12)	0.0049 (16)
O3	0.0397 (9)	0.0281 (9)	0.0290 (9)	0.0003 (7)	−0.0060 (8)	0.0002 (8)
O4	0.0359 (9)	0.0253 (9)	0.0263 (9)	0.0058 (7)	−0.0030 (8)	−0.0008 (7)
C6	0.0594 (18)	0.0432 (15)	0.0348 (16)	−0.0087 (13)	−0.0015 (15)	−0.0039 (15)
C7	0.0299 (13)	0.0299 (14)	0.0328 (15)	−0.0100 (10)	0.0023 (11)	−0.0017 (11)
C9	0.0229 (11)	0.0222 (11)	0.0360 (14)	−0.0025 (9)	0.0036 (11)	0.0003 (11)
C10	0.0351 (14)	0.0278 (13)	0.0451 (17)	0.0018 (11)	−0.0008 (13)	0.0036 (12)
C8	0.0342 (13)	0.0230 (13)	0.0338 (15)	−0.0024 (10)	0.0076 (11)	−0.0051 (10)
N2	0.0656 (17)	0.0331 (14)	0.0447 (16)	0.0041 (12)	0.0035 (14)	−0.0136 (12)
O9	0.114 (3)	0.0271 (14)	0.072 (2)	−0.0078 (15)	0.006 (2)	−0.0089 (15)
O10	0.121 (3)	0.063 (2)	0.069 (3)	0.0254 (19)	0.045 (2)	−0.0107 (17)
O9A	0.061 (10)	0.025 (9)	0.055 (11)	0.004 (8)	−0.020 (9)	−0.025 (8)
O10A	0.083 (11)	0.044 (10)	0.086 (13)	0.002 (9)	0.024 (10)	−0.038 (10)
O5	0.0301 (9)	0.0286 (9)	0.0366 (10)	−0.0006 (7)	0.0008 (8)	−0.0001 (8)
O6	0.0381 (9)	0.0234 (9)	0.0311 (11)	0.0006 (6)	−0.0008 (9)	−0.0033 (8)
C11	0.0358 (15)	0.0351 (15)	0.060 (2)	−0.0033 (12)	−0.0018 (14)	0.0147 (13)
C12	0.0280 (12)	0.0233 (12)	0.0477 (16)	0.0037 (9)	−0.0076 (13)	0.0081 (14)
C14	0.0441 (15)	0.0231 (13)	0.0402 (18)	0.0078 (11)	−0.0053 (12)	−0.0057 (12)
C15	0.068 (2)	0.0368 (15)	0.0434 (19)	0.0009 (13)	−0.0029 (16)	−0.0134 (14)
C13	0.0383 (18)	0.0222 (14)	0.0512 (19)	−0.0025 (13)	−0.0102 (15)	−0.0034 (13)
N3	0.0388 (19)	0.0424 (18)	0.088 (2)	−0.0136 (14)	−0.002 (2)	−0.0241 (19)
O11	0.0459 (19)	0.082 (3)	0.150 (5)	−0.0126 (18)	−0.016 (3)	−0.050 (3)
O12	0.0668 (16)	0.0290 (14)	0.117 (3)	−0.0038 (13)	0.0069 (18)	−0.0255 (16)
C13A	0.0383 (18)	0.0222 (14)	0.0512 (19)	−0.0025 (13)	−0.0102 (15)	−0.0034 (13)
N3A	0.0388 (19)	0.0424 (18)	0.088 (2)	−0.0136 (14)	−0.002 (2)	−0.0241 (19)
O11A	0.051 (8)	0.081 (9)	0.087 (10)	−0.017 (7)	−0.035 (8)	−0.021 (8)
O12A	0.068 (10)	0.064 (9)	0.086 (10)	−0.043 (8)	−0.006 (8)	−0.017 (8)

### Geometric parameters (Å, º)

**Table:**

Co1—O1	1.8635 (16)	C9—C10	1.493 (3)
Co1—O5	1.8686 (17)	C10—H10A	0.9800
Co1—O6	1.869 (2)	C10—H10B	0.9800
Co1—O4	1.8694 (16)	C10—H10C	0.9800
Co1—O2	1.8722 (17)	C8—N2	1.461 (3)
Co1—O3	1.8721 (19)	N2—O9A	1.208 (14)
O1—C2	1.261 (3)	N2—O10A	1.216 (15)
O2—C4	1.263 (3)	N2—O9	1.217 (4)
C1—C2	1.512 (3)	N2—O10	1.235 (4)
C1—H1A	0.9800	O5—C12	1.267 (3)
C1—H1B	0.9800	O6—C14	1.264 (3)
C1—H1C	0.9800	C11—C12	1.501 (4)
C2—C3	1.403 (4)	C11—H11A	0.9800
C3—C4	1.412 (4)	C11—H11B	0.9800
C3—N1	1.465 (3)	C11—H11C	0.9800
C4—C5	1.495 (4)	C12—C13	1.399 (5)
C5—H5A	0.9800	C12—C13A	1.432 (13)
C5—H5B	0.9800	C14—C13	1.412 (5)
C5—H5C	0.9800	C14—C13A	1.439 (13)
N1—O8	1.224 (4)	C14—C15	1.499 (4)
N1—O7	1.224 (3)	C15—H15A	0.9800
O3—C7	1.270 (3)	C15—H15B	0.9800
O4—C9	1.264 (3)	C15—H15C	0.9800
C6—C7	1.485 (4)	C13—N3	1.466 (4)
C6—H6A	0.9800	N3—O12	1.204 (4)
C6—H6B	0.9800	N3—O11	1.230 (5)
C6—H6C	0.9800	C13A—N3A	1.482 (16)
C7—C8	1.405 (4)	N3A—O12A	1.201 (18)
C9—C8	1.395 (4)	N3A—O11A	1.202 (18)
			
O1—Co1—O5	87.03 (7)	O4—C9—C10	114.4 (2)
O1—Co1—O6	87.82 (8)	C8—C9—C10	122.9 (2)
O5—Co1—O6	94.70 (7)	C9—C10—H10A	109.5
O1—Co1—O4	174.00 (8)	C9—C10—H10B	109.5
O5—Co1—O4	88.92 (7)	H10A—C10—H10B	109.5
O6—Co1—O4	88.11 (7)	C9—C10—H10C	109.5
O1—Co1—O2	95.40 (7)	H10A—C10—H10C	109.5
O5—Co1—O2	176.10 (8)	H10B—C10—H10C	109.5
O6—Co1—O2	88.46 (8)	C9—C8—C7	127.2 (2)
O4—Co1—O2	88.89 (7)	C9—C8—N2	116.8 (2)
O1—Co1—O3	88.84 (8)	C7—C8—N2	116.1 (2)
O5—Co1—O3	88.69 (8)	O9A—N2—O10A	121.6 (13)
O6—Co1—O3	175.11 (7)	O9—N2—O10	123.0 (3)
O4—Co1—O3	95.49 (7)	O9A—N2—C8	116.2 (9)
O2—Co1—O3	88.30 (8)	O10A—N2—C8	121.6 (10)
C2—O1—Co1	126.46 (16)	O9—N2—C8	119.0 (3)
C4—O2—Co1	126.32 (16)	O10—N2—C8	117.9 (3)
C2—C1—H1A	109.5	C12—O5—Co1	124.92 (17)
C2—C1—H1B	109.5	C14—O6—Co1	124.96 (18)
H1A—C1—H1B	109.5	C12—C11—H11A	109.5
C2—C1—H1C	109.5	C12—C11—H11B	109.5
H1A—C1—H1C	109.5	H11A—C11—H11B	109.5
H1B—C1—H1C	109.5	C12—C11—H11C	109.5
O1—C2—C3	122.6 (2)	H11A—C11—H11C	109.5
O1—C2—C1	112.4 (2)	H11B—C11—H11C	109.5
C3—C2—C1	125.0 (2)	O5—C12—C13	121.6 (3)
C2—C3—C4	126.1 (2)	O5—C12—C13A	123.8 (6)
C2—C3—N1	118.1 (2)	O5—C12—C11	113.6 (3)
C4—C3—N1	115.8 (2)	C13—C12—C11	124.7 (3)
O2—C4—C3	122.5 (2)	C13A—C12—C11	121.7 (6)
O2—C4—C5	113.8 (2)	O6—C14—C13	121.3 (3)
C3—C4—C5	123.6 (2)	O6—C14—C13A	123.6 (7)
C4—C5—H5A	109.5	O6—C14—C15	114.1 (3)
C4—C5—H5B	109.5	C13—C14—C15	124.6 (3)
H5A—C5—H5B	109.5	C13A—C14—C15	121.0 (7)
C4—C5—H5C	109.5	C14—C15—H15A	109.5
H5A—C5—H5C	109.5	C14—C15—H15B	109.5
H5B—C5—H5C	109.5	H15A—C15—H15B	109.5
O8—N1—O7	124.2 (3)	C14—C15—H15C	109.5
O8—N1—C3	119.0 (3)	H15A—C15—H15C	109.5
O7—N1—C3	116.8 (3)	H15B—C15—H15C	109.5
C7—O3—Co1	126.27 (17)	C12—C13—C14	126.7 (3)
C9—O4—Co1	126.28 (17)	C12—C13—N3	118.9 (3)
C7—C6—H6A	109.5	C14—C13—N3	114.3 (3)
C7—C6—H6B	109.5	O12—N3—O11	124.7 (3)
H6A—C6—H6B	109.5	O12—N3—C13	118.1 (3)
C7—C6—H6C	109.5	O11—N3—C13	117.2 (4)
H6A—C6—H6C	109.5	C12—C13A—C14	122.1 (11)
H6B—C6—H6C	109.5	C12—C13A—N3A	113.4 (11)
O3—C7—C8	122.1 (2)	C14—C13A—N3A	124.4 (12)
O3—C7—C6	114.9 (2)	O12A—N3A—O11A	120.5 (19)
C8—C7—C6	123.0 (2)	O12A—N3A—C13A	118.3 (17)
O4—C9—C8	122.6 (2)	O11A—N3A—C13A	121.2 (15)
			
O5—Co1—O1—C2	−177.2 (2)	C7—C8—N2—O9	−120.7 (4)
O6—Co1—O1—C2	−82.3 (2)	C9—C8—N2—O10	−120.0 (4)
O2—Co1—O1—C2	5.9 (2)	C7—C8—N2—O10	61.0 (4)
O3—Co1—O1—C2	94.1 (2)	O1—Co1—O5—C12	65.7 (2)
O1—Co1—O2—C4	1.0 (2)	O6—Co1—O5—C12	−21.9 (2)
O6—Co1—O2—C4	88.7 (2)	O4—Co1—O5—C12	−109.90 (19)
O4—Co1—O2—C4	176.8 (2)	O3—Co1—O5—C12	154.58 (19)
O3—Co1—O2—C4	−87.6 (2)	O1—Co1—O6—C14	−64.51 (19)
Co1—O1—C2—C3	−10.3 (4)	O5—Co1—O6—C14	22.32 (19)
Co1—O1—C2—C1	170.89 (18)	O4—Co1—O6—C14	111.09 (19)
O1—C2—C3—C4	7.7 (4)	O2—Co1—O6—C14	−159.97 (19)
C1—C2—C3—C4	−173.7 (3)	Co1—O5—C12—C13	8.8 (4)
O1—C2—C3—N1	−174.7 (3)	Co1—O5—C12—C13A	20.7 (10)
C1—C2—C3—N1	3.9 (4)	Co1—O5—C12—C11	−169.52 (16)
Co1—O2—C4—C3	−3.4 (4)	Co1—O6—C14—C13	−9.6 (4)
Co1—O2—C4—C5	178.68 (17)	Co1—O6—C14—C13A	−21.4 (10)
C2—C3—C4—O2	−0.3 (4)	Co1—O6—C14—C15	171.42 (18)
N1—C3—C4—O2	−178.0 (3)	O5—C12—C13—C14	12.8 (5)
C2—C3—C4—C5	177.4 (3)	C13A—C12—C13—C14	−93 (4)
N1—C3—C4—C5	−0.3 (4)	C11—C12—C13—C14	−169.1 (3)
C2—C3—N1—O8	133.5 (3)	O5—C12—C13—N3	−171.0 (3)
C4—C3—N1—O8	−48.7 (4)	C13A—C12—C13—N3	83 (4)
C2—C3—N1—O7	−46.8 (4)	C11—C12—C13—N3	7.0 (5)
C4—C3—N1—O7	131.1 (3)	O6—C14—C13—C12	−12.4 (5)
O1—Co1—O3—C7	173.7 (2)	C13A—C14—C13—C12	94 (4)
O5—Co1—O3—C7	86.68 (19)	C15—C14—C13—C12	166.5 (3)
O4—Co1—O3—C7	−2.11 (19)	O6—C14—C13—N3	171.4 (3)
O2—Co1—O3—C7	−90.83 (19)	C13A—C14—C13—N3	−83 (4)
O5—Co1—O4—C9	−84.85 (18)	C15—C14—C13—N3	−9.8 (5)
O6—Co1—O4—C9	−179.59 (18)	C12—C13—N3—O12	130.7 (4)
O2—Co1—O4—C9	91.91 (19)	C14—C13—N3—O12	−52.7 (6)
O3—Co1—O4—C9	3.73 (19)	C12—C13—N3—O11	−46.7 (7)
Co1—O3—C7—C8	0.4 (3)	C14—C13—N3—O11	129.9 (5)
Co1—O3—C7—C6	179.31 (17)	O5—C12—C13A—C14	−12.7 (19)
Co1—O4—C9—C8	−3.6 (3)	C13—C12—C13A—C14	68 (4)
Co1—O4—C9—C10	174.29 (15)	C11—C12—C13A—C14	178.3 (9)
O4—C9—C8—C7	0.8 (4)	O5—C12—C13A—N3A	168.2 (9)
C10—C9—C8—C7	−176.8 (2)	C13—C12—C13A—N3A	−111 (5)
O4—C9—C8—N2	−178.0 (2)	C11—C12—C13A—N3A	−0.8 (15)
C10—C9—C8—N2	4.3 (3)	O6—C14—C13A—C12	13.1 (19)
O3—C7—C8—C9	0.9 (4)	C13—C14—C13A—C12	−67 (4)
C6—C7—C8—C9	−178.0 (2)	C15—C14—C13A—C12	179.4 (10)
O3—C7—C8—N2	179.7 (2)	O6—C14—C13A—N3A	−167.9 (11)
C6—C7—C8—N2	0.9 (4)	C13—C14—C13A—N3A	112 (5)
C9—C8—N2—O9A	108.5 (17)	C15—C14—C13A—N3A	−1.6 (18)
C7—C8—N2—O9A	−70.5 (17)	C12—C13A—N3A—O12A	51 (2)
C9—C8—N2—O10A	−63 (2)	C14—C13A—N3A—O12A	−128 (2)
C7—C8—N2—O10A	118 (2)	C12—C13A—N3A—O11A	−130.2 (19)
C9—C8—N2—O9	58.3 (4)	C14—C13A—N3A—O11A	51 (2)

### Hydrogen-bond geometry (Å, º)

**Table:**

*D*—H···*A*	*D*—H	H···*A*	*D*···*A*	*D*—H···*A*
C1—H1*B*···O7^i^	0.98	2.33	3.087 (4)	134
C11—H11*B*···O12^ii^	0.98	2.55	3.240 (4)	128
C10—H10*C*···O5^iii^	0.98	2.46	3.433 (3)	176
C15—H15*C*···O3^iv^	0.98	2.57	3.542 (4)	174

Symmetry codes: (i) −*x*+1, *y*, *z*−1/2; (ii) *x*, −*y*+1, *z*−1/2; (iii) −*y*+1, *x*−1/2, *z*+1/4; (iv) *x*, *y*, *z*+1.
